# A systematic review of factors contributing to partners’ negative birth experiences

**DOI:** 10.1186/s12884-026-08889-6

**Published:** 2026-03-14

**Authors:** Rebecca Hunter, Leonardo De Pascalis, Pauline Slade

**Affiliations:** 1 Department of Primary Care and Mental Health, University of Liverpool, Bedford Street South, Liverpool, L69 7ZA UK; 2 Department of Psychological Science, University of Liverpool, Bedford Street South, Liverpool, L69 7ZA UK; 3 Department of Psychology, University of Bologna, Viale Berti Pichat 5, Bologna, 40127 Italy

**Keywords:** Partners, Birth experience, Negative birth experience, Birth trauma

## Abstract

**Background:**

Most partners attend the birth of their infant. Partners can be psychologically impacted by the birth they witness. It is important to understand what may contribute to this experience being appraised as negative, so that services can develop support for partners. This systematic review aimed to explore partners’ reported negative experiences of childbirth. The objective was to synthesise qualitative literature on aspects of experience that contributed to partners appraising childbirth as negative. ENTREQ framework for reporting the synthesis of qualitative health research was used to report findings.

**Methods:**

A qualitative meta-synthesis design was adopted. Searches were conducted using the databases PsycINFO, Medline, CINAHL, PubMed, EMCare, and Web of Science using search terms relating to partners and childbirth experiences. The Critical Appraisal Skills Programme (CASP) was used to assess the quality of the included studies.

**Results:**

Sixteen studies were included in the review. Themes generated from the meta-synthesis were ‘high negative emotionality during the birth’, ‘unprepared/not going as expected’, ‘communication from healthcare professionals’, ‘exclusion’, ‘clarity of role’, and ‘perceived low control’.

**Conclusions:**

Partners experienced intense negative emotions, particularly when births did not go as expected, and these feelings were amplified by poor communication, exclusion, and uncertainty about their role. Feeling unprepared, both practically and emotionally, and perceiving a lack of control were central contributors to negative experiences. Partners’ experiences were shaped not only by the birth itself but also by the quality of healthcare interactions and the clarity of their own role, with cultural context further influencing expectations and emotional responses.

**Supplementary Information:**

The online version contains supplementary material available at 10.1186/s12884-026-08889-6.

## Background

The field of perinatal psychology expanded greatly in recent years since National Health Service (NHS) England provided funding to develop and expand specialist perinatal and maternal mental health services across the United Kingdom (UK; [1, 2]. The National Institute for Health and Care Excellence (NICE) states that women should have timely access to specialist evidence-based psychological interventions for mental health difficulties experienced in the perinatal period [3]. Currently, these guidelines only recommend assessment and signposting for partners [3, 4].

Childbirth is a significant event in a person’s life that can be associated with a range of positive and negative emotions [5, 6]. In the UK, 91% of partners attend the birth of their first child and their involvement in labour and childbirth is widely encouraged and promoted within maternity services [7]. Partner involvement in labour and childbirth was suggested to have positive benefits for the woman giving birth and for the partner’s transition to parenthood [8, 9]. Although childbirth was generally considered safe in the UK, the experience of trauma is subjective and it is well recognised that labour and birth with or without complications can be experienced as traumatic by mothers and their partners [10–18].

Partners’ negative experiences of birth are often associated with complicated or adverse events, such as premature birth, emergency caesarean section, and postpartum haemorrhage [19–21]. Partners reported that a lack of support from healthcare professionals also contributes to distress following difficult birth experiences [16]. As a result, partners can often feel strong emotions including anxiety, helplessness, fear, and feeling out of control during childbirth [16].

A recent meta-analysis examined prevalence rates of birth-related Post-Traumatic Stress Disorder (PTSD) in partners [22]. The results indicated prevalence rates ranging from 0% to 7.2% with an overall estimated prevalence of 1.2%. In partners, the prevalence was found to be higher in routine samples (2.1%) than in targeted groups (0.8%), such as those whose infant had an admission to the neonatal intensive care unit, their partner delivered via emergency caesarean, or they had a history of trauma.

Given the emerging evidence around partners’ negative experiences of childbirth [16, 23], it is important to better understand which aspects of the experience lead to an overall negative appraisal of the birth. Subjective appraisals of birth as negative increased the likelihood of experiencing the birth as traumatic [17], which is linked to an increased risk of developing PTSD [13, 17] potentially impacting on the parent-infant relationship [24]. Therefore, gaining a better understanding of the factors that contribute to negative appraisals of childbirth is crucial.

An increased understanding of what contributes to a negative birth experience in partners could help services develop and improve how they screen and support these partners, which, in turn, could lead to improved experiences and outcomes for partners, women, and their families.

### Aim

This systematic review aimed to explore partners’ reported negative experiences of childbirth. The objective was to synthesise qualitative literature on aspects of experience that contributed to partners appraising childbirth as negative. A qualitative methodology was adopted due to the limited number of large-scale quantitative studies on partners’ experiences of birth. Therefore, the focus of the current review was to better understand the key aspects of partners’ experiences through qualitative accounts.

## Methods

### Definitions

In the current review, the term ‘partner’ was defined as a father or birth partner in a co-parenting role. A negative birth experience was defined as the partner being present for the birth and later describing the experience as negative, traumatic, and/or stressful, or expressing dissatisfaction with the birth experience, when recalling the birth in the postnatal period. The postnatal period was defined as any time point after the birth, with no upper limit.

### Design

The design utilised was a qualitative meta-synthesis [25]. The design involved six steps: (1) defining the research question and selection criteria, (2) searching for and selecting the studies via relevant databases (3) quality assessing the studies, (4) extracting and presenting the formal data, (5) analysing and interpreting the data, and (6) writing the synthesis [25–29]. Enhancing transparency in reporting the synthesis of qualitative research (ENTREQ) framework for reporting the synthesis of qualitative health research was used to report findings [30].

### Search method

Searches were performed within PsycINFO, Medline, Cumulative Index to Nursing and Allied Health Literature (CINAHL), PubMed, EMCare, and Web of Science. Search terms used were (Male* or men or men’s or man or man’s or father* or dad or dads or partner* or co-parent* or paternal* or parent* or parental) AND (Birth* or childbirth or labour or labor or deliver*) AND (Trauma* or traumatic or negative* or experienc* or distress* or stress* or PTSD or post-traumatic stress disorder or complicated or complications) AND (Qualitative or qualitatively). In addition, Medical Subject Headings (MeSH) were used as part of the search strategy to increase the accuracy of the searches [31]. Relevant MeSH were generated by reviewing literature in the area. The MeSH terms used within the search strategy were (Parturition or Psychology or Pregnancy or Obstetric Labor Complications or Humans or Natural Childbirth or Delivery, Obstetric). Searches were pre-planned and aimed to identify all available studies.

A Population, Interest, Context (PICo) [32] matrix was developed to outline inclusion and exclusion criteria for the review. Studies were included if they were published in English and reported qualitative data from partners in a co-parenting role, aged over 18, who were present at the labour and/or birth of their infant. Studies relating to stillbirth, premature birth, or multiple births were excluded. Studies were excluded if their primary data was not related to partners. Articles from peer-reviewed journals were included and case studies, conference papers, literature reviews, and discussion papers were excluded. Reference lists were also hand-searched for relevant studies. Full inclusion and exclusion criteria are available on request from the primary author.

Ten per cent of titles and abstracts (*n* = 1380) were cross-checked for inclusion/exclusion by an independent rater. Inter-rater agreement was 100% (κ = 1). After titles and abstracts were screened, full texts of the included titles were screened for inclusion/exclusion. Again, 10% of the full texts (*n* = 4) were also cross-checked for inclusion/exclusion by the same independent rater. The inter-rater agreement indicated moderate agreement (75%, κ = 0.5). Any disagreements were discussed with the wider research group, and an agreement was reached. The lower agreement observed at the full-text stage reflected the inherent complexity of applying nuanced inclusion criteria to qualitative studies, particularly in relation to the interpretation of ‘negative experience.’ All disagreements were resolved through discussion within the full research team.

Searches were completed in September 2022 and repeated in April 2024. The repeated search conducted in April 2024 did not identify any additional eligible papers. Given the substantial interval between searches, we employed citation tracking and expert consultation to confirm that no major relevant publications were overlooked. Themes following the analysis were generated by RH and discussed and refined in discussion with PS and LDP.

### Search results

**Fig. 1:**
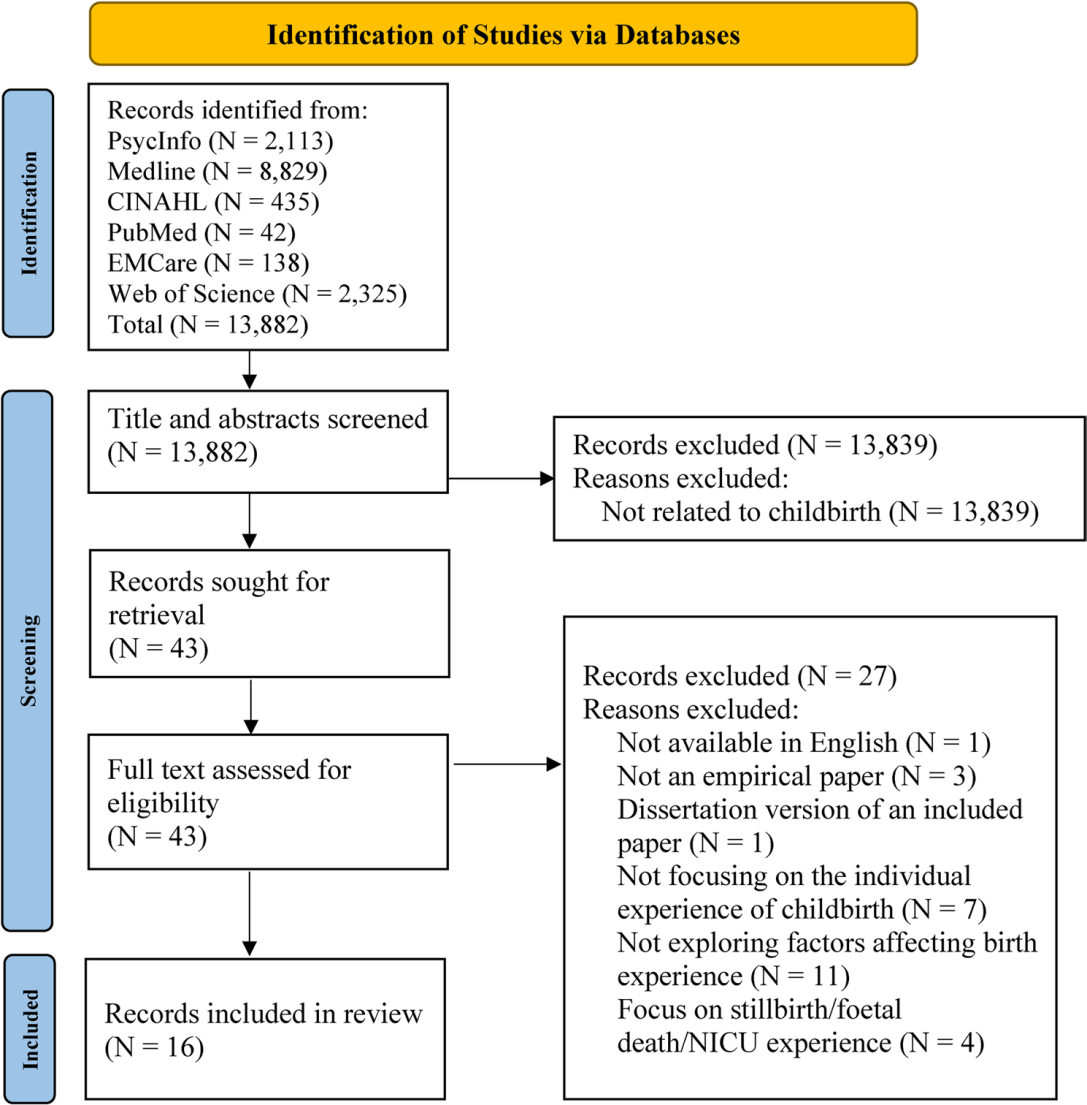
PRISMA flow diagram of screening and selection process [33]

A total of 13,882 studies were identified. The Preferred Reporting Items for Systematic Reviews and Meta-Analyses (PRISMA) chart can be seen in Fig. 1 [33]. After applying the inclusion and exclusion criteria 16 studies remained [34–49]. Hand-searching of reference lists did not generate any additional studies to be included. Included studies can be seen in Table 1.

**Table 1:** Characteristics of studies included in the meta-synthesis

Author	Title	Country	Age of child/Time scale	Measurement	Analysis	Themes
Daniels, Arden-Close, & Mayers, (2020) [34]	Be quiet and man up: A qualitative questionnaire study into fathers who witnessed their partner’s birth trauma.	UK	Within 10 years of birth.	Questionnaire. 20 open-ended questions relating to the birth experience.	Thematic Analysis	(1) Fathers’ understanding of the experience; (2) life after birth trauma; (3) the support fathers received vs. what they wanted.
Elmir & Schmied (2022) [35]	A qualitative study of the impact of adverse birth experiences on fathers.	Australia & New Zealand	4.5 months to 20.5 years.	Face-to-face, telephone, and email interviews	Interpretive Phenomenological Analysis	(1) Worst experience of my life; (2) negotiating my place; (3) communicating with health professionals; (4) growing stronger or falling apart.
Etheridge & Slade (2017) [36]	“Nothing’s actually happened to me.”: The experiences of fathers who found childbirth traumatic.	UK	2 months to 6 years.	Semi-structured interview Impact of Event Scale	Template Analysis	(1) Experience of birth; (2) impact on self; (3) “nothing’s actually happened to *me*”; (4) putting it “in a box”; (5) relationships; (6) desire for resolution; (7) what may have helped and when.
Hinton, Locock, Knight (2014) [37]	Partner experiences of ‘‘near-miss’’ events in pregnancy and childbirth in the UK: A qualitative study.	UK	14 weeks to “several years”.	Semi-structured interview	Interpretive Phenomenological Analysis	(1) Experiences in hospital; (2) communication; (3) father’s/partner’s emotional recovery
Inglis, Sharman, Reed (2016) [38]	Paternal mental health following perceived traumatic childbirth.	Australia	No cut-off. 3 participants reported their child to be over 12 years old.	Online open-ended survey Semi-structured interview	Thematic Analysis	(1) Standing on the side-line; (2) witnessing trauma; (3) unknown territory, and (4) the aftermath: dealing with it.
Johnson (2002) [39]	An exploration of men’s experience and role at childbirth.	UK	Questionnaires- within 60 h after birth. Interviews- within one week after birth.	Semi-structured interview Pregnancy Outcome Survey	Thematic Analysis Descriptive Statistics	(1) Reaction to the birth; (2) men’s perceived role at childbirth; (3) men’s reasons for being present at childbirth; (4) are men prepared for their role during, and the experience of, childbirth?
Kululanga, Malata, Chirwa, Sundby (2012) [40]	Malawian fathers’ views and experiences of attending the birth of their children: A qualitative study.	Malawi	Within 2 years of birth.	Semi-structured interview	Content Analysis	(1) Positive experiences; (2) negative experiences; (3) reflection and (4) resolutions.
Lindberg & Engstrom (2013) [41]	A qualitative study of new fathers’ experiences of care in relation to complicated childbirth.	Sweden	1.5-3 months after birth.	Semi-structured interview.	Content Analysis	(1) Struggling to be recognised by care staff as a partner in the family; (2) Feeling scared and uncared for during acute situations; (3) Appreciating the opportunity to participate in care and becoming a family; (4) Needing continued care.
Lwanga, Atuyambe, Sempewo, Lumala, & Byaruhanga (2017) [42]	An exploratory study of men’s companionship, perceptions and experiences during pregnancy and delivery in Uganda.	Uganda	Not reported.	Semi-structured interview	Content Analysis	(1) Feelings about attending childbirth; (2) responsibilities during childbirth; (3) positive experiences; (4) negative experiences about childbirth.
Messner (2018) [43]	Overview of transition to fatherhood in the context of caesarean birth.	USA	Not reported.	Semi-structured interview	Interpretive Phenomenological Analysis	(1) Expecting a natural childbirth; (2) ongoing communication breakdowns; (3) riding an emotional rollercoaster; (4) moving to a sense of relief and peace; (5) awakenings to the challenges of being a father; (6) resolving the memory and moving on.
Nicholls & Ayers (2007) [44]	Childbirth-related post-traumatic stress disorder in couples: a qualitative study.	UK	Within 3 months to 10 years of the birth.	Semi-structured interview	Thematic Analysis	(1) Birth factors; (2) quality of care; (3) effects on relationship with a partner; (4) effects on relationship with the child.
Sapkota, Kobayashi, Toshio, Takase, & Miyuki (2012) [45]	Husbands’ experiences of supporting their wives during childbirth in Nepal.	Nepal	Within 7 days of the birth.	Semi-structured interview	Thematic Analysis	(1) Being positive towards attendance; (2) hesitation; (3) poor emotional reactions; (4) being able to support; (5) the need to be mentally prepared and (6) enlightenment.
Sengane & Cur (2009) [46]	The experience of black fathers concerning support for their wives/partners during labour.	South Africa	2 days postpartum.	Semi-structured interview	Content Analysis	(1) The body; (2) intellect; (3) emotion; (4) volition; (5) Spirit Aspect
Talley (2018) [47]	First-time fathers’ perspectives on pregnancy, birth, and fatherhood.	USA	Within 6 months of the birth.	Semi-structured interview	Interpretive Phenomenological Analysis	(1) Mood swings; (2) watching the belly grow during pregnancy; (3) it (labour) was a life-changing experience; (4) fear of something happening to the baby or mom; (5) becoming more selfless and responsible; (6) fear of making a mistake as a father; (7) and the difficulties of fatherhood.
White (2007) [48]	You cope by breaking down in private: fathers and PTSD following childbirth.	New Zealand	No cut-off. Range: 2 weeks to 32 years after birth.	Verbal (direct to the researcher or submitted on tape) or written (letter or email)	Content Analysis	(1) It’s not a spectator sport; (2) it’s about being included; (3) it’s sexual scarring; (4) it’s toughing it out.
Zwedberg, Bjerkan, Asplund, Ekeus, & Hjelmstedt (2015) [49]	Fathers’ experiences of a vacuum extraction delivery – a qualitative study.	Sweden	One month after birth.	Semi-structured interview	Content Analysis	(1) Affected but helpless; (2) Wish to be involved; (3) Anxious observer; (4) Turbulent feelings; (5) Thoughts about consequences

### Quality appraisal

The methodological quality of papers was assessed using the Critical Appraisal Skills Programme (CASP) [50]. The CASP was the most widely used tool for quality appraising qualitative research [51]. No study was excluded due to poor quality. The quality appraisal of included studies can be seen in Table 2.

**Table 2:** Appraisal of Qualitative Studies using the Critical Appraisal Skills Programme (CASP)

	Daniels, et al. (2020) [34]	Elmir & Schmied (2022) [35]	Etheridge & Slade (2017)* [36]	Hinton et al. (2014) [37]	Inglis, Sharman, Reed (2016) [38]	Johnson (2002)* [39]	Kululanga et al. (2012) [40]	Lindberg & Engstrom (2013) [41]	Lwanga et al. (2017) [42]	Messner (2018) [43]	Nicholls & Ayers (2007) [44]	Sapkota et al. (2012) [45]	Sengane & Cur (2009) [46]	Talley (2018) [47]	White (2007) [48]	Zwedberg et al. (2015) [49]
Was there a clear statement of the aims of the research?	Y	Y	Y	Y	Y	Y	Y	Y	Y	Y	Y	Y	Y	Y	Y	Y
Is the qualitative methodology appropriate?	Y	Y	Y	Y	Y	Y	Y	Y	Y	Y	Y	Y	Y	Y	Y	Y
Was the research design appropriate to address the aims?	Y	Y	Y	Y	Y	Y	Y	Y	Y	Y	Y	Y	Y	Y	Y	Y
Was the recruitment strategy appropriate to the aims?	Y	Y	Y	Y	Y	Y	Y	Y	Y	Y	Y	Y	Y	Y	Y	Y
Was the data collected in a way that addressed the research issue?	Y	Y	Y	Y	Y	Y	Y	Y	Y	Y	Y	Y	Y	Y	Y	Y
Has the relationship between the researcher and participants been adequately considered?	Y	Y	N	N	Y	N	N	Y	Y	N	N	N	N	Y	Y	N
Have ethical issues been taken into consideration?	Y	Y	Y	Y	Y	Y	Y	Y	Y	N	Y	Y	Y	Y	Y	Y
Was the data analysis sufficiently rigorous?	Y	Y	Y	Y	Y	Y	Y	Y	Y	N	Y	Y	Y	Y	Y	Y
Is there a clear statement of findings?	Y	Y	Y	Y	Y	Y	Y	Y	Y	Y	Y	Y	Y	Y	Y	Y

*Note. Y: Yes; N: No; X: Cannot tell*

Overall, the quality of the included studies was considered high. Only one study was considered of lower quality compared to other studies included in the review, due to a lack of coherence between data collection, analysis, and interpretation, and inadequate substantiation of data in participant quotes [43]. However, other elements of the study, such as a clear statement of aims and findings, as well as an appropriate recruitment strategy, were considered high quality. The CASP tool identified nine studies which did not consider the relationship between the author and the participants [36, 37, 39, 40, 43–46, 49]. The authors’ lack of stated reflexivity that considers the contextual, intersecting relationship between themselves and their participants limited the credibility and the contextual understanding of the findings [52].

### Data abstraction and synthesis

The first step of data abstraction and synthesis involved reading each study several times to become familiar with participant quotes, metaphors, key concepts, and interpretations [25, 29]. Key data from the studies, such as participant demographics and study methodology, were also extracted and are available in supplementary materials. The second step involved coding the studies line by line at a descriptive level, identifying key concepts and meanings within the data [25, 29].

The third step involved grouping and categorizing the codes into themes, comparing them across studies to identify similarities and differences, and establishing the extent to which findings from one study differed from another [25, 29]. The final step required going beyond the content and descriptions of the included studies to achieve a higher level of interpretation and understanding of the intended concept. This step involved analysing, reviewing, and refining the generated themes, to ensure they were meaningful interpretations of the included studies [25, 53]. The included studies employed a range of qualitative methodologies, including interpretative phenomenological analysis (IPA), thematic analysis, and content analysis. To integrate these diverse approaches, a thematic translation method was used: sub-themes identified in individual studies were first coded inductively within the context of their original analytic framework and then compared across studies to identify conceptual similarities and differences. These comparable sub-themes were then grouped into broader meta-themes through iterative discussion and comparison across studies. For example, individual codes such as “lack of updates during emergency,” “confusion about medical procedures,” and “feeling excluded from decisions” were translated into the broader meta-theme “Communication from healthcare professionals.” Higher-order interpretations were reached by examining how these meta-themes related to one another and to the overarching research question. Disagreements in theme boundaries or naming were resolved through discussion within the research team, referring to original data extracts to achieve a final consensus. To support transparency in how findings from different qualitative traditions were integrated, a thematic translation approach was adopted that prioritised conceptual meaning over methodological origin. During synthesis, decisions about how to translate themes across studies were guided by their conceptual relevance and explanatory value rather than by methodological hierarchy or frequency counts. No studies were given greater weight solely on the basis of methodology; instead, all included studies were appraised for quality, and their contribution to the synthesis was determined by the richness, clarity, and relevance of their findings.

These sub-themes were then grouped into broader meta-themes that captured shared meanings across methodological boundaries. The thematic translation method ensured that findings from different qualitative methodologies could be synthesised.

### Reflexivity

Reflexivity in research enhanced rigour by considering how the researchers’ experiences and interests affected the stages of the research [25–27]. The author conducted the initial synthesis independently, with evidence for the themes being discussed with the wider research team. The findings were therefore analysed through the lens of the author’s experiences. The author identified as a female researcher from a high-income country (HIC), with no personal experience of birth but with clinical experience in perinatal psychology services. This background may have heightened sensitivity to emotional and psychological aspects of partners’ accounts and influenced the framing of findings through a lens informed by professional practice.

To mitigate these influences, the analysis was grounded closely in the primary data and checked against direct participant quotations. Reflexive notes were used throughout coding and theme development to question assumptions and ensure interpretations remained aligned with participants’ intended meanings.

Interpretation was also discussed with the wider research team, which included a male member with recent exposure to a birth experience within a HIC healthcare context. While this perspective contributed to broadening interpretive insight, we acknowledged that no members of the team had lived or professional experience of providing or receiving maternity care in Low- and Middle-Income Countries (LMIC).

## Results

### Study characteristics

Five of the included studies were conducted in the UK, three were conducted across Australia and New Zealand, and two were conducted in each of the United States of America and Sweden. One study was completed in each of Malawi, Uganda, South Africa, and Nepal. There was a total of 308 partners across the 16 studies. Timescales since the negative birth experience ranged from 2 days to 32 years postpartum.

Three studies examined traumatic childbirths only [34, 36, 38]. Two of these studies relied on participants self-identifying as having perceived the childbirth experience to be traumatic [34, 38], while the third study defined traumatic childbirth as partners who experienced feelings of intense fear, helplessness or horror at some point during the birth [36]. Five studies looked at births where there were complications or adverse outcomes [35, 37, 41, 43, 49]. Three of these studies looked specifically at certain modes of delivery, with two exploring unplanned caesarean Sects [41, 43]. and one unplanned vacuum extractions [49]. Three studies looked specifically at first-time fathers’ experiences [36, 45, 47].

Four of the studies were completed in countries where partners’ attendance at childbirth is much lower compared to HIC, and cultural norms and practices discourage men’s attendance in the birthing room (e.g. Malawi, Nepal, South Africa, and Uganda) [40, 42, 45, 46]. These studies were still included in the review to better understand any differences and similarities that may have existed about the review question across cultures, and therefore culture and context were considered throughout the results.

### Sample characteristics

The sample characteristics of the included studies are available in supplementary materials. All studies reported experiences from male partners. One study had a female partner in a same-sex relationship in their sample [37]. In studies where marital status was reported (*n* = 8), 89.3% were males married to female gestational parents (*n* = 150). Eight studies did not report the marital status of participants. Ethnicity or racial background was reported in nine studies and reflected a diverse range of ethnic backgrounds. The age of participants across all studies ranged from 18 to 69. Employment status was only reported in some of the studies (*n* = 6). Where this information was reported, there was a range of employment statuses reported including unemployed, full-time students, manual labour jobs, and professional roles. Three studies reported findings from first-time co-parents only and the remaining studies (*n* = 13) reported findings from both multiparous and primiparous partners. The range cannot be reported as three studies did not report exactly how many children the partners had.

Overall, the sample characteristics were predominantly individuals who identified as heterosexual males married to female gestational parents. The sample was diverse in terms of age range, the number of children the partner had, and employment status. A range of ethnicities were represented, and culture and context were considered throughout the results and discussion.

### Themes

Six themes were generated from the meta-synthesis. Themes were ‘high negative emotionality during the birth’, ‘unprepared/not going as expected’, ‘communication from healthcare professionals’, ‘exclusion’, ‘clarity of role’ and ‘perceived low control’. Table 3 provides an overview of how the themes generated in this review map onto the themes reported within each of the included studies. This mapping was intended to enhance transparency and demonstrate the analytic coherence between the included literature and the themes developed through the synthesis.

**Table 3:** Relationship between themes in included studies and themes generated by meta-synthesis

	High negative emotionality during the birth	Unprepared/ not going as expected	Communication from healthcare professionals	Exclusion	Clarity of role	Perceived low control
Daniels, Arden-Close, & Mayers, (2020) [34]	“Merely a passenger”“Not about me”	“Nothing can prepare you for it”“Prenatal support”	“Mixed experiences with staff”“Not about me”	“Not about me”	“Not about me”	“Merely a passenger”
Elmir & Schmied (2022) [35]	“Worst experience of my life” “Negotiating my place: Communication with health professionals”	“Worst experience of my life”	“Negotiating my place: Communication with health professionals”	“Negotiating my place: Communication with health professionals”	“Negotiating my place: Communication with health professionals”	
Etheridge & Slade (2017) [36]	“Rapid changes in events and environment” “Fears of death” “Trying to ‘keep it together’” “Helplessly watching a catastrophe unfold”	“A rollercoaster: rapid changes in events and environment”	“Isolation/abandonment: left in a room” Staff and system responses: communication”	“Isolation/abandonment: left in a room” “Being sent away”		
Hinton, Locock, Knight (2014) [37]	“Communication”	“Powerlessness and exclusion” “Witnessing”	“Powerlessness and exclusion” “Communication”	“Powerlessness and exclusion” “Communication”		
Inglis, Sharman, Reed (2016) [38]		“Witnessing trauma: Unknown territory” “Being unprepared”	“Standing on the side-line” “Not knowing”	“Standing on the side-line” “Not knowing”		“Out of control”
Johnson (2002) [39]	“Are men prepared for their role during, and the experience of, childbirth?”	“Are men prepared for their role during, and the experience of, childbirth?”			“Men’s perceived role at childbirth”	
Kululanga, Malata, Chirwa, Sundby (2012) [40]	“Shame and embarrassment” “Helplessness and unprepared”	“Shame and embarrassment” “Helplessness and unprepared”	“Helplessness and unprepared” “Health worker-male partner tension”	“Exclusion from decision-making process”		“Shame and embarrassment”
Lindberg & Engstrom (2013) [41]	“Being scared and uncared for in the acute situation”		“Struggling to be recognised by care staff as a partner in the family”	“Struggling to be recognised by care staff as a partner in the family”		
Lwanga, Atuyambe, Sempewo, Lumala, & Byaruhanga (2017) [42]	“Anxious and frightening” “Painful”	“Anxious and frightening” “Unplanned reactions”			“Supportive role” “Decision maker”	
Messner (2018) [43]	“Emotional roller coaster”	“Pre-delivery expectations”	“Waiting without knowing” “Trust in staff”			“Loss of control”
Nicholls & Ayers (2007) [44]	“Negative emotions in labour”	“Expectations not being met”	“Lack of choice or lack of involvement in decision making” “Information provision” “Staff factors”	“Lack of choice or lack of involvement in decision making”		“Perceived lack of control”
Sapkota, Kobayashi, Toshio, Takase, & Miyuki (2012) [45]	“Poor emotional reactions” “Being able to support”	“Poor emotional reactions” “Being able to support” “Being mentally prepared”			“Being able to support”	
Sengane & Cur (2009) [46]	“Emotion” “Volition”					
Talley (2018) [47]	“Adverse perceptions and experiences of childbirth”	“Adverse perceptions and experiences of childbirth”				
White (2007) [48]	“It’s not a spectator sport” “It’s about being included”	“It’s not a spectator sport”		“It’s about being included”	“It’s not a spectator sport”	
Zwedberg, Bjerkan, Asplund, Ekeus, & Hjelmstedt (2015) [49]	“Anxious observer” “Turbulent feelings”		“Wish to be involved”		“Affected but helpless”	

### High negative emotionality during the birth

Partners expressed feeling a range of strong emotions during the birth experience in 15 of the studies [34–37, 39–49]. These emotions included fear, frustration, confusion, anger, shock, powerlessness, and helplessness. The quality of studies in this theme was considered high.

Fear and powerlessness were expressed in studies investigating self-reported traumatic birth experiences and related to not knowing what was happening to the mother and infant and whether they were safe [34, 35, 43]. Seeing their partner in pain and/or distress and feeling the need to be “strong” for their partner contributed to feelings of helplessness and powerlessness to do anything to relieve the pain [36, 39, 40, 42, 45–49].

Hinton et al., examined obstetric complications/unexpected emergencies during birth, partners reported feeling intense fear and profound shock at what was happening around them [37]. In this study, the time since the “near-miss” birth ranged from less than one year to over 10 years. Some participants did not have any lasting psychological effects from the experience; however, some were deeply affected, even years later, with the recall still being vivid. Witnessing medical procedures, such as the administration of epidural and use of forceps and ventouse, was also experienced as traumatic by partners and evoked emotions, such as terror [47–49].

Embarrassment was an emotion mentioned in studies from LMIC; vaginal examinations were experienced as shameful and embarrassing in a study completed in Malawi [40]. These emotions may have been linked to the men feeling unprepared for what to expect from the birth due to it not being culturally accepted for them to join antenatal classes or appointments. Similarly, in Nepal, partners felt embarrassed and hesitant to be in the delivery room, due to cultural ideas around it being a woman’s arena [45]. There appeared to be similarities in the experiences of terror and fear across the cultures included in the review, however, embarrassment, hesitation, and shame appeared to be unique to the studies where men supporting their partner’s during labour is seen as “taboo” [40, 42, 45, 46]. Three of these studies, however, did not consider reflexivity and their own relationship to the topic, meaning that there may be a level of bias within the findings [40, 45, 46].

### Unprepared/not as expected

The ‘Unprepared/not as expected’ theme reflected partners’ experiences and reports of feeling unprepared for the birth experience and comprises evidence from 13 studies [34–40, 42–45, 47, 48]. Again, the quality of the studies within this theme was considered high.

In the studies that were examining self-reported traumatic birth experiences, most partners reported feeling unprepared for the birth [34, 38, 44]. Preparation was considered to be attendance at antenatal classes and through information provided by healthcare professionals [34, 40]. Antenatal classes were described as “too positive” and tended to focus on “standard deliveries” and the mother’s experience and did not provide information about possible complications or what to expect during the birth from a partners’ perspective [16, 34, 38, 64]. As a result, some partners felt unprepared or unaware of how to support the mother during birth [42].

Other studies investigating first-time co-parents, found that they generally reported expecting a “normal uneventful birth” and had little or no knowledge about what to expect [36, 42, 45, 47]. However, feeling unprepared was not limited to just first-time co-parents, and was a relevant theme to studies that included partners who already had children, indicating that every birth is unique and can be experienced differently from prior births that partners may have been present for [34, 35, 37–40, 42, 44, 48].

During the birth, not knowing whether things were normal (e.g. amount of blood loss), lots of healthcare professionals being present in the room, and worried facial reactions of healthcare professionals, contributed to a sense of terror and concern and were experiences that the partners did not expect [35, 36, 40, 42, 47].

Studies looking specifically at obstetric complications found that partners’ experiences varied, possibly due to the wide range of reasons for which an obstetric complication or emergency can occur. For some, there was a long “build-up” during the pregnancy and/or labour, and for others, the complications happened very quickly [37]. Nonetheless, partners did not feel prepared for things “going wrong” and experienced fear and a sense of powerlessness in response [37, 48]. Additionally, some partners went into the birth expecting a ‘natural’ delivery, so when their infant was born via an unplanned intervention such as an emergency caesarean section, they found the experience difficult to process because it differed so markedly from their expectations [43].

In the studies completed in cultures where men are generally not encouraged to attend antenatal classes and childbirth, participants felt similarly unprepared [40, 42, 45]. Participants reported feeling unprepared due to either not being told what to expect by healthcare professionals during antenatal visits, or not attending antenatal classes with the mother. For example, in studies conducted in Malawi [40] and Nepal [45], the participants had not attended antenatal classes with the mother and, as a result, felt unprepared about what to expect during childbirth and how to provide support to the mother during labour. The partners reported feeling frustrated by this lack of knowledge and, after the birth experience, felt that they should be included in antenatal and birth preparation in the future [45]. Two of these studies did not consider their own experiences and the influence this may have had on the interpretation of the data, which is a limitation that must be considered.

### Communication from healthcare professionals

‘Communication from healthcare professionals’ relates to communication between healthcare professionals and partners during birth and includes evidence from 10 studies [34–38, 40, 41, 43, 44, 49]. The theme included a study of poorer quality [43] and six that did not consider reflexivity in their findings [36, 37, 40, 43, 44, 49].

Calm and clear communication from healthcare professionals was seen as protective and was experienced positively by the partners [34], particularly in the context of obstetric emergencies or unexpected complications [37, 41, 49]. Concerning emergency caesarean sections, partners felt that better communication from healthcare professionals around what was happening would have reduced their feelings of stress during the experience and could have supported their engagement in decision-making [41, 43]. One of these studies, however, was of poorer quality due to a lack of coherence between data collection, analysis, and interpretation, and a lack of participant quotes to evidence themes [43].

Some partners reported staff communication changing when things “went wrong” and that they were not provided with adequate information about what was happening [35, 37, 43, 44, 49]. Others felt that communication was poor throughout the birth experience [40]. In the context of obstetric emergencies, the quality of communication from healthcare professionals was considered important in shaping how partners later appraised the experience [37]. In six studies, partners reported feeling like “second-class citizens” and were left for long periods before being given any news on what was happening to the mother and infant [36–38, 41, 43, 44]. In some cases, there was an actual physical separation from partners and the mother and infant (due to having to go to the operating theatre for an emergency caesarean section) without knowing what was happening, and healthcare professionals did not communicate vital information to them [36–38, 41].

Being provided with consistent information from a known midwife/obstetrician was considered to make a positive difference in the partners’ experiences [35, 41, 49]. Unfortunately, continuity of care is not always possible, and, for some partners, the lack of midwife continuity was detrimental to their experience of care and birth [34, 43].

The experience of communication from healthcare professionals appeared to be similar across cultures. However, a crucial distinction lay in *expectations* of communication, which strongly influenced whether poor communication was experienced as distressing [40, 46]. In LMIC studies, such as those from Malawi, partners described healthcare professionals as “experts” and themselves and the mother as “passive recipients of care,” and they did not expect to be consulted on decision-making [41]. As these expectations aligned with the limited communication they received, the lack of information was not generally perceived as distressing, reflecting trust in the professionals and the process. In contrast, partners in studies from HIC typically expected frequent updates, inclusion in decision-making, and clear explanations during the birth. When these expectations were not met, they described significant frustration, helplessness, and distress. The findings therefore suggested that it was not simply the absence of communication itself, but the gap between what was expected and what was delivered, that shaped negative appraisals, a pattern more pronounced in HIC contexts.

However, the authors in two of these studies did not consider how their own cultural experiences, assumptions, and beliefs, may have influenced the research, which has to be considered when interpreting their findings [40, 46].

### Exclusion

The “Exclusion” theme related to partners feeling as if they were “standing on the side-line” and excluded at various points during the antenatal period and the birth experience and was comprised of evidence from nine studies [34–38, 40, 41, 44, 48]. The quality of the included studies in the theme was considered high.

Participants reported feeling excluded at antenatal classes and that, ideally, classes should have promoted inclusion from the outset [34]. Being present for antenatal classes and appointments but only feeling like a “witness” to the conversations contributed to feelings of exclusion and that healthcare professionals did not consider or value the partner’s perspectives or experiences [34, 35, 48]. Partners felt as if they were not listened to, their questions were not answered, and they were not taken seriously [34, 48].

During obstetric emergencies, partners felt that they were excluded from what was happening and not involved in decision-making [37, 41, 44, 48]. In White’s [48] study, some of the recollections of witnessing birth trauma were from over 20 years previously, but participants reflected that the experience remained vivid, and the sense of exclusion was disempowering and depersonalising.

Similarly to the communication from healthcare professionals’ theme, the main differences in the experience of exclusion across cultures appeared to be centred around the expectations of exclusion. In Kululanga et al., [40], partners did not expect to be involved in the decision-making and were accepting of that lack of involvement. Healthcare professionals made all the decisions, and the couples were “told what to do”; the absence of involvement was not experienced or appraised as traumatic or challenging but viewed as culturally acceptable and normal.

The themes of ‘Exclusion’ and ‘Clarity of role’ were closely related, with feelings of exclusion often directly contributing to role ambiguity. When partners were not included in conversations, decision-making, or practical care, they were left uncertain about how they could meaningfully contribute, reinforcing perceptions of being on the outskirts. Conversely, when partners had a clearly defined and acknowledged role, they reported feeling more included. While they are distinct themes, these findings suggested that exclusion can act as a driver of role ambiguity, and that improving partner inclusion may simultaneously enhance role clarity.

### Clarity of role

The ‘Clarity of Role’ theme relates to how clear partners felt their role during birth was and encompasses evidence from seven studies [34, 35, 39, 42, 45, 48, 49]. The quality of the included studies in the theme was considered high. Three studies did not consider reflexivity in their findings [39, 45, 49].

Some participants described feeling unclear about their role during birth and feeling like “a spare wheel” [34]. In addition, partners largely described feeling as if they were not acknowledged by healthcare professionals. For those who did feel acknowledged, it was felt as if their role was solely to support the mother, rather than as an active participant in the birth of their infant [34, 48].

In births where there were unexpected obstetric complications, some partners saw their role as “advocates” for the mother and infant. These partners, therefore, felt distressed when they were not included in decision-making around the care of the mother and infant during the birth [35]. In births that ended with vacuum extraction, partners initially felt like active participants and highly involved in the birth, but their role changed to that of an observer when the decision for vacuum extraction was made by health professionals [49]. Partners did not feel involved in the decision-making for vacuum extraction and felt that they were not provided with adequate information.

In terms of cultural differences, it appeared that partners in Nepal and Uganda felt more confident in assuming a role during the birth, despite feeling unprepared for the event. First-time co-parents in Nepal felt confident in providing emotional support to the mother and in acting as a liaison between their wife and healthcare professionals [45]. Providing physical support to the mother is a role they felt less confident in. In the Lwanga et al., [42] study, partners also seemed confident in assuming a role during birth. Roles included a supportive role, providing physical support (e.g. massage, picking things up) and comfort to their partners and the role of “decision-maker”. These studies were from cultures where male-gendered roles are seen as providers and decision-makers, which may have contributed to their confidence in their role during birth.

### Perceived low control

Perceived levels of control during birth were mentioned in five studies [34, 38, 40, 43, 44]. Three of these studies were looking specifically at traumatic birth experiences and found that a perceived lack of control contributed to the experience being appraised as traumatic [34, 38, 44]. Three of the studies included in the theme were considered lower quality due to the authors not considering reflexivity in their findings, limiting the credibility of the research through lack of acknowledgement about how their subjectivity may have influenced the findings [40, 43, 44].

There was a perception of either no control at all or that control was taken away from partners due to the escalating emergency. Where there was a perceived high level of control, partners reflected more positively about the birth and may have been a protective factor against partners developing mental health difficulties postnatally [34].

No cultural differences were noted across the studies. A perceived low sense of control over the situation contributed to feelings of fear and helplessness across cultures [40].

The themes identified in this review were presented as distinct but co-occurring aspects of partners’ experiences; the qualitative data did not support establishing a hierarchy or directional relationships between themes, and they were therefore interpreted as parallel rather than causal constructs.

## Discussion

The current systematic review synthesised qualitative evidence to identify the key factors that contribute to partners appraising childbirth negatively. High levels of negative emotionality, such as terror, helplessness, and stress, were central to these appraisals. A recent literature review exploring partners’ mental health and perceived social support during complicated childbirth supported these findings [16]. That review similarly reported that partners experienced complex negative emotions during labour, particularly when obstetric complications occur, and highlighted how poor relationships and communication with healthcare professionals can intensify these feelings, further aligning with the current findings [16].

In addition, the current review found the birth ‘not going as expected’ contributed to the birth being appraised as negative by the partner. The nature of this ‘unpreparedness’ was multi-faceted, encompassing; informational gaps, particularly around potential complications, medical interventions, and their likely emotional impact; limited practical preparation, such as skills in coping, advocacy, or supporting decision-making during the birth; and insufficient emotional readiness for the intensity and unpredictability of labour. Several studies described antenatal classes as being “too positive” or overly focused on idealised birth narratives, which left partners feeling unprepared when complications arose. Similar findings were suggested in the literature for women. For example, in a recent systematic review, Webb et al., [6] found that a large discrepancy between expectations of the birth and the actual birth experience had a negative impact on women’s perceptions of their birth experience and increased their risk of developing postnatal PTSD. Addressing these dimensions of preparedness in antenatal education by including realistic scenario-based discussions, opportunities for couples to discuss roles, and explicit exploration of emotional coping strategies, might have helped increase preparedness. Where appropriate, antenatal classes could have expanded the breadth and depth of coverage around birth, to be more realistic and better prepare partners for various scenarios [34]. This finding was supported by research demonstrating that antenatal education for partners can have positive outcomes, including reduced anxiety and improved satisfaction with the birth experience [54–57]. This ‘expectation-experience gap’ holds important implications for intervention design: efforts to enhance communication quality may prove most efficacious when coupled with strategies to align partner expectations through culturally informed antenatal preparation that addresses the realities of diverse birth trajectories.

Poor communication from healthcare professionals, was also identified as an important elements that contributed to partners negative appraisals and reactions. Partners often reported that healthcare professionals could have communicated more information about what was happening to them, which would have had the potential to reduce the experience of strong negative emotions, such as stress. This finding was supported by a previous review which found that effective communication with maternity healthcare professionals helped to build rapport and trust during shared decision-making during pregnancy and childbirth [58] and another systematic review that found good communication between parents and maternity healthcare professionals mediated birth experience when assisted vaginal delivery was required [59].

Clarity of role and perceived level of control were also aspects that were identified as influencing how the partner appraised the birth experience. Partners who found the birth experience to be traumatic experienced either no control or that control was taken away from them during the delivery. This finding was supported by previous literature that found partners of pregnant women did not anticipate having a sense of control in relation to the childbirth experience and that they felt uncertain around their role in the process [60–62]. Considering these findings, it may be that when births go “as expected” or without any unexpected obstetric complications, a partner’s role as supporter and coach feels comfortable and clear, however, when there are complications, the role of the partner may become less clear, and a role of advocacy and decision-making may become more important.

One of the most distinctive contributions of this review is the explicit cross-cultural results. While previous literature has focused almost exclusively on partner experiences in HIC, this review integrates evidence from both HIC and LMIC and identified how cultural norms actively shape partners’ expectations, emotional responses, and appraisals of birth experiences. This finding moves the field beyond merely describing “poor communication” or “role ambiguity” as universal problems, to showing that the emotional impact of these factors is strongly moderated by context and cultural expectations.

The cultural differences noted in the current systematic review are a novel contribution to the literature. There were similarities across cultures in terms of partners feeling unprepared for the birth experience and experiencing high negative emotionality during birth (e.g., terror, fear). However, hesitation, embarrassment, and shame appeared unique to the LMIC included in the review. In some LMIC, assuming a role during birth (e.g., providing comfort, advocacy) felt easier, contrasting with partners from HIC who often felt unclear about their role. Experiences of communication from healthcare professionals were broadly consistent across cultures, but their appraisal differed: partners in LMIC did not expect open communication or involvement in decision-making and were therefore not distressed by exclusion, whereas those expecting communication found the lack to be distressing. A particularly important nuance concerned expectations of communication from healthcare professionals. In LMIC contexts, such as Malawi, partners tended to view healthcare professionals as unquestioned experts and themselves as passive recipients of care and therefore did not expect regular updates or involvement in decision-making. As a result, limited communication was not generally perceived as distressing and often aligned with cultural norms. By contrast, partners in HIC studies expected frequent, transparent updates and active participation; when these expectations were not met, they reported frustration, helplessness, and distress. This finding indicated that the emotional impact of poor communication was shaped more by the gap between expected and actual communication than by its absence alone, a finding particularly relevant for understanding cross-cultural differences in birth experience appraisal.

These cultural differences were supported by previous research indicating that in settings where male attendance is not culturally accepted, there is tension between men’s wishes and health system practices [20, 21]. Partners often reported feeling excluded and unprepared despite wanting involvement [20, 21, 63, 64]. Across cultures, increased antenatal preparation involving both partners may improve preparedness and potentially reduce intense negative emotions during birth.

The sample characteristics of the studies represented the experiences of heterosexual male partners of female gestational parents, limiting generalisability of findings. Experiences of same-sex partners, non-cohabiting partners, and those in non-traditional family structures might have differed. These partners may have faced distinct challenges, such as navigating heteronormative assumptions within maternity care and reduced acknowledgement of their role. The absence of these perspectives in the current evidence base represented an important gap and highlights the need for future research to explore the birth experiences of diverse partner groups to ensure inclusive, equitable maternity care. The sample was diverse in age, parity, employment, and ethnicity. Reflexivity could have been stronger in several studies, but overall quality was high, supporting the robustness of findings.

The current review adds to the evidence base of partners’ experiences of being present for childbirth. Clear communication and involving partners in decision-making were important in supporting partners during childbirth. Partners wanted to feel included throughout pregnancy and birth, particularly in the context of complications, but currently often feel excluded.

Recent reviews published after the completion of our search further illustrate the rapidly evolving nature of this field. Schmitt et al. (2022) conducted a scoping review of partners’ experiences in HIC settings and identified themes relating to intense emotions, the role of support, staff interactions, and the transition to fatherhood [65]. The scope of their review differs to the current review as they excluded births that were complicated or perceived as traumatic. Nonetheless, these areas show clear conceptual overlap with the themes generated in our synthesis, reinforcing the relevance of the relational, emotional, and contextual dimensions highlighted in our findings. McNab et al. (2022) similarly reviewed factors influencing fathers’ childbirth experiences and their implications for postnatal mental health, underscoring the importance of support, communication, and expectations, elements that align closely with the themes identified in the current review [66]. While these more recent reviews differ in scope and methodological approach, they collectively support and extend the patterns observed in the current synthesis.

### Strengths and limitations

The strengths of this review include the diversity of the sample across age, parity, employment status, and ethnicity. The inclusion of studies from multiple cultural contexts enhanced the applicability of the findings and represents a novel contribution to the literature on partners’ experiences of childbirth. However, there was an absence of an intersectional analysis, meaning the review did not fully consider how culture, gender, and socio-economic status may interact to shape partners’ experiences; future research should explore these intersecting influences in greater depth.

As with all qualitative evidence syntheses, the findings reflect the specific populations, settings, and circumstances represented in the included studies and therefore lack generalisability from a statistical perspective. Although qualitative insights are context-rich, they might not have fully captured the experiences of partners in settings or cultures not represented in the included studies. A recognised methodological challenge in qualitative evidence synthesis concerns the rigour and consistency of searching for and selecting qualitative studies, which might have increased the risk of missing relevant evidence [67]. To minimise this risk, we used a comprehensive search strategy across multiple databases, applied clear inclusion and exclusion criteria, and reviewed reference lists of included studies. These steps align with recommended approaches for enhancing rigour in qualitative reviews and reducing the likelihood of overlooking eligible studies [68]. Additional limitations include the exclusion of grey literature and the restriction to English-language publications, both of which might have introduced bias and limit the breadth of perspectives captured.

An additional limitation is that studies focusing on premature birth, stillbirth, or neonatal intensive care unit experiences were excluded; therefore, the findings are not generalisable to partners whose negative experiences arise from these high-risk and highly specialised clinical situations, where the nature and intensity of distress might have differed substantially. Additionally, most included studies focused on heterosexual, cohabiting male partners, with limited diversity in relation to gender identities and relationship structures. Emerging research suggests that non-binary, lesbian, gay, bisexual, transgender, and queer (LGBTQ+), and non-cohabiting partners might have encountered additional or distinct barriers during childbirth, including heightened exclusion, assumptions about their legitimacy as a support person, and a lack of recognition within heteronormative maternity systems [69]. As this is an evolving area of research, future studies should prioritise more diverse samples to better understand how gender identity, sexual orientation, and family structure shape partners’ experiences of childbirth and their interactions with maternity services.

A further limitation relates to the positionality of the research team. All authors are based in HIC contexts, and none have lived or professional experience of providing or receiving maternity care in low- or middle-income countries. This was particularly relevant given the cross-cultural considerations raised in the review. The findings should therefore be interpreted with awareness that the research team’s perspectives were shaped by HIC contexts, and that additional insights from researchers or service users with LMIC experience may have further enriched the analysis.

### Implications for practice

The recommendations outlined below emerged predominantly from HIC contexts and reflect underlying assumptions about partner involvement that may not be universally applicable or culturally appropriate. In LMIC settings where male attendance during childbirth remain contested, culturally complex, or contextually constrained, these recommendations should be adapted judiciously through meaningful consultation with local stakeholders, taking into account prevailing cultural norms, resource availability, and women’s preferences regarding partner involvement.

Partners wanted realistic, inclusive antenatal education and meaningful involvement during maternity care. Evidence from multiple studies demonstrated that partner-focused antenatal classes could reduce anxiety, improve confidence, and enhance satisfaction relating to their maternity experience [54–57]. Incorporating partners in discussions about possible complications, clarifying their potential roles, and maintaining open communication during birth might have helped mitigate negative appraisals. Healthcare professionals should consider proactively engaging partners in both routine and emergency contexts. Although evidence supports partner-focused antenatal education generally, the specific intervention components that effectively target the modifiable factors remain under-investigated and warrant prioritisation in future intervention research.

The findings of this review, together with supporting literature, highlight several areas where changes in practice may help improve partners’ experiences of childbirth and reduce the likelihood of negative appraisals. To support more inclusive, informed, and culturally attuned care for partners, the following recommendations are proposed:

1. Strengthen antenatal education for partners by providing realistic preparation for a range of birth outcomes and potential complications, helping partners feel more informed and emotionally prepared.
2. Support couples to discuss and define the partner’s intended role during labour, enabling clearer expectations and reducing uncertainty during the birth.
3. Promote inclusive and consistent communication across the maternity system, ensuring partners receive timely information and are appropriately involved in decision-making where culturally and clinically appropriate.
4. Embed cultural awareness and contextual sensitivity throughout maternity care, recognising that expectations of involvement and communication vary across cultural settings and that recommendations may need to be adapted for partners and families in different contexts.

These recommendations aim to enhance partners’ sense of involvement, preparedness, and emotional safety during childbirth, while acknowledging that the research team’s HIC perspectives may shape the interpretation and applicability of these suggestions.

### Implications for future research

Future research should examine how specific antenatal education interventions for partners affect birth appraisals and postnatal mental health. Cross-cultural comparative studies are warranted to explore how cultural expectations shape partners’ experiences. Further work could explore the impact of structured role-definition for partners and evaluate their impact on perceived control, preparedness, and satisfaction with the birth experience.

## Conclusions

This systematic review aimed to explore partners’ reported negative experiences of childbirth. Findings highlighted that partners experienced intense negative emotions, particularly when births did not go as expected, and these feelings were amplified by poor communication, exclusion, and uncertainty about their role. A lack of practical and emotional preparation, combined with a perceived loss of control, emerged as central drivers of negative appraisals. Importantly, partners’ experiences were shaped not only by the clinical events of birth but also by the quality of their interactions with healthcare professionals and the extent to which their involvement was supported. Cultural context further influenced expectations, shaping how partners interpreted and responded to the birth environment. Collectively, these findings highlight the need for maternity systems to recognise partners as integral participants in the birth experience. Interventions that strengthen antenatal preparation, promote inclusive and timely communication, and clarify the partner’s role have the potential to reduce distress, enhance emotional safety, and improve the overall childbirth experience for families.

## Supplementary Information

Supplementary Material 1.

## Data Availability

All data generated or analysed during this study are included in this published article.

## Abbreviations

CASP: Critical Appraisal Skills Programme
CINAHL: Cumulative Index to Nursing and Allied Health Literature
HIC: High-Income Countries
LMIC: Low-and Middle-Income Countries
MeSH: Medical Subject Headings
NHS: National Health Service
NICE: National Institute for Health and Care Excellence
PICo: Population, Interest, Context
PRISMA: Preferred Reporting Items for Systematic Reviews and Meta-Analyses
PTSD: Post-Traumatic Stress Disorder
UK: United Kingdom
