# Supplementary material for: A systematic review of factors contributing to partners’ negative birth experiences
Source: BMC Pregnancy Childbirth. 2026 Mar 14;26:447. doi: 10.1186/s12884-026-08889-6 (PMC13101201; doi:10.1186/s12884-026-08889-6)
Supplement: Supplementary file 1 — Supplementary Material 1. [file 12884_2026_8889_MOESM1_ESM.pdf]

Supplementary Materials 1

Study Sample Characteristics

Author	Participants	N	Age of partner (range)	Gender	Marital status	Number of children (range)	Ethnicity or race	Single or multiple births	Employment status
Daniels, Arden-Close, & Mayers, (2020)	Males aged 18+, residing in the UK who were present for childbirth that they perceived as traumatic (that did not result in loss of life).	61	24-51	Male N = 61	Not reported	1-4	Not reported	Single infant N = 60 Twins N = 1	Not reported
Elmir & Schmied (2022)	Males who were present for childbirth.	17	24-48	Male N = 17	Married N = 13 Single N = 1 In de facto relationships N = 3	1-3	Not reported	Not reported	Not reported
Etheridge & Slade (2017)	Males aged 18+ who were present for childbirth and answered yes to "At some point during the childbirth I	11	27-45	Male N = 11	Married N = 7 Cohabiting N = 3 Engaged N = 1	1	Not reported	Not reported	Employed N = 11

Supplementary Materials 1

Study Sample Characteristics

Author	Participants	N	Age of partner (range)	Gender	Marital status	Number of children (range)	Ethnicity or race	Single or multiple births	Employment status
	experienced feelings of intense fear, helplessness or horror”.								
Hinton, Locock, Knight (2014)	Women and partners who experienced a life-threatening obstetric emergency	35 women, 11 partners	21-40	Women/mothers N = 35 Fathers/partners N = 11 (10 male and 1 female)	Not reported	Not reported	White British N = 42 British Pakistani N = 1 White Australian N = 2 White Israeli N = 1	Not reported	Professional N = 20 Other non-manual N = 13 Skilled manual N = 4 Unskilled manual N = 2 Other (such as housewife or student) N = 7
Inglis, Sharman, Reed (2016)	Males who were present for childbirth and perceived it as traumatic.	69 survey responses<br><br>7 interviews	The range was not reported. Mean = 36.71	Male N = 69	Married N = 58 In de facto relationships N = 4 Single N = 3 Divorced N = 2 Separated N = 1	1->5	Not reported	Not reported	Not reported

Supplementary Materials 1

Study Sample Characteristics

Author	Participants	N	Age of partner (range)	Gender	Marital status	Number of children (range)	Ethnicity or race	Single or multiple births	Employment status
					Engaged N = 1				
Johnson (2002)	Males who were present for childbirth.	53 completed surveys<br>20 interviews	18-56	Male N = 53	Not reported	1-5	Not reported	Single infant N = 51<br>Twins N = 2	Not reported
Kululanga, Malata, Chirwa, Sundby (2012)	Males who were present for childbirth in the last 2 years.	20	29-50	Male N = 20	Married N = 20	1+ (upper number not reported)	Chewa N = 6<br>Ngoni N = 4<br>Tumbuka N =4<br>Mang'anja N =2<br>Yao N = 2<br>Lomwe N = 2	Not reported	Paid up jobs N = 17<br>Self-employed N = 3
Lindberg & Engstrom (2013)	First-time fathers who had witnessed their partner's emergency caesarean section.	8	24-44	Male N = 8	Not reported	1-3	Not reported	Not reported	Not reported
Lwanga, Atuyambe, Sempewo, Lumala, &	Males aged 18+ who were present for childbirth.	16	22-40	Male N = 16	Married N = 16	1+ (upper number not reported)	Ganda N = 8<br>Nyankole N = 2<br>Others N = 6	Not reported	Not reported

Supplementary Materials 1

Study Sample Characteristics

Author	Participants	N	Age of partner (range)	Gender	Marital status	Number of children (range)	Ethnicity or race	Single or multiple births	Employment status
Byaruhanga (2017)									
Messner (2018)	First-time fathers where the childbirth was an unexpected caesarean delivery.	4	Not reported	Male N = 4	Not reported	Not reported	Not reported	Not reported	Not reported
Nicholls & Ayers (2007)	Couples where at least one partner had clinically significant symptoms of childbirth-related PTSD.	12 (6 couples)	26-50	Male N = 6<br>Female N = 6	Married N = 12	1-2	White European N = 11<br>White Australian N = 1	Not reported	Not reported
Sapkota, Kobayashi, Toshio, Takase,	First-time fathers who were present for childbirth.	12	21-32	Male N = 12	Married N = 12	1	Brahman N = 3<br>Chhetri N = 3<br>Newar N = 2	Not reported	Private business employee N = 5<br>Government employee N = 4

Supplementary Materials 1

Study Sample Characteristics

Author	Participants	N	Age of partner (range)	Gender	Marital status	Number of children (range)	Ethnicity or race	Single or multiple births	Employment status
& Miyuki (2012)							Tamang/Magar/ Gurung N = 3 Minor N = 1		Driver/carpenter N = 2 Farmer N = 1
Sengane & Cur (2009)	Group 1- Black males who were present for childbirth. Group 2- control- Black males who were not present for childbirth.	10	Not reported	Male N = 10	Not reported	Not reported	Black N = 10	Not reported	Not reported
Talley (2018)	First-time fathers who were present for childbirth.	12	18-34	Male N = 10	Married N = 12	1	African American N = 3 Caucasian N = 9	Not reported	Employed N = 10 Unemployed N = 1 Student N = 1
White (2007)	Males who were present for childbirth.	21	30-69	Male N = 21	Not reported	1-4	Caucasian N = 18 Pacific Island N = 2 Maori N = 1	Not reported	Not reported

Supplementary Materials 1

Study Sample Characteristics

Author	Participants	N	Age of partner (range)	Gender	Marital status	Number of children (range)	Ethnicity or race	Single or multiple births	Employment status
Zwedberg, Bjerkan, Asplund, Ekeus, & Hjelmstedt (2015)	Fathers who were present for childbirth via vacuum extraction.	10	25-67	Male N = 10	Not reported	1-3	Swedish N = 8<br>Mexican N = 1<br>Iraqi N = 1	Not reported	Not reported
